# Farewell Address to the Class of 1896

**Published:** 1896-07

**Authors:** A. C. Bernays


### Abstracts and Selections.

### Farewell Remarks to the Class of '96.

The following are the farewell remarks delivered at the annual banquet tò the alumni of the Marion-Sims College of Medicine, at the St. Nicholas Hotel, St. Louis, Mo., April 2nd, 1896, by Prof. A. C. Bernays:

GENTLEMEN:—Permit me to read you the following quotations and thoughts that I have put together for you instead of making an attempt to respond to the toast proposed, which I feel unable to do:

Remember that "having fine sentiments is a poor substitute for being a man!"

Remember that "dignity is no more the sign of wisdom than a paper collar is of a shirt." All quacks wear silk high-hats and make a show of dignity. The scientific attainments of doctors are almost exactly in the inverse ratio to their show of dignity and pose. Ignorance is most easily hidden under the cloak of dignity and by keeping a close mouth.

"Remember to judge people by what they do, not by their sentiments—especially yourself."

Remember that in order to freely and cheerfully recognize merit in others, you must be worthy and meritorious yourself.

"Remember that you may have your best friends among those who disagree with you. Men can disagree with their heads and agree in their hearts."

"Remember that prejudice hurts the one who cherishes it much more than the one against whom it is aimed."

Remember that it makes no difference at all what a man believes but a great deal what he knows.

Remember that the way to conquor prejudice is to live it down. Do not discuss it with others, waste no thought on it yourself.

Remember that it is brave to be in the minority. That is where the strong usually are. Weak natures can not stand alone, but must lean on the majority.

Remember that it is the nature of science to ignore authority, to look away from it, to pursue its own course in order that it may arrive at the highest and most important truths without prejudice.

"In your lives follow nothing but the beacon light of reason. It will lead you to the truth."

"Remember that schools are the churches, universities the cathedrals where the only true religion can be found, and that the only possible savior of the human race is science."

Remember that after to-night you must give up text-books in order to study nature. The only way in which you will be able to advance the interests of our profession will be by adding to our knowledge; the only way in which you will be able to do that, will be by using your trained senses in observing facts and by recording your observations and reflections in a scientific medical journal.

Finally, gentlemen, remember "there is no darkness but ignorance," and remember in your toilsome professional career to shed as much light along your course as you may be able to create or to reflect. Remember my oft-repeated commandment: Scientific truths must be freely given away, they are priceless, and one who trades in them is unworthy of the ware. Give them to others just as you have received them from me at this college from which you have graduated to-night. I hope that the wants of your bodies and the hunger of your minds may be satisfied, so that you will be happy enough to make others happy.

